# A Recently Recognized and Underdiagnosed Entity of Splenic Diffuse Red Pulp Small B-Cell Lymphoma: A Report of Two Cases

**DOI:** 10.7759/cureus.107946

**Published:** 2026-04-29

**Authors:** Siham Belkadi, Imane El Boutahiri, Hari Oumayma, Ghizlane Erradi, Safae Regragui

**Affiliations:** 1 Clinical Hematology Department, Mohammed VI University Hospital, Tangier, MAR; 2 Anatomopathology Department, Mohammed VI University Hospital, Tangier, MAR

**Keywords:** primary splenic lymphoma, sdrpl, splenic diffuse red pulp small b-cell lymphoma, splenomegaly, villous lymphocytes

## Abstract

Splenic diffuse red pulp small B-cell lymphoma (SDRPL) is an extremely rare entity newly individualized in the WHO 2022 classification, following provisional recognition in 2008. It remains underrecognized in routine practice. Its diagnosis is challenging because it overlaps with other splenic lymphomas with villous lymphocytes, particularly splenic marginal zone lymphoma (SMZL) and hairy cell leukemia variants, with only a few cases reported due to recent recognition. We present two patients illustrating their distinctive diagnostic and therapeutic phenotypes. Case 1 was a 65-year-old woman diagnosed in 2017 with SMZL and treated with rituximab, cyclophosphamide, hydroxydaunorubicin (doxorubicin), oncovin (vincristine), and prednisone (or prednisolone) (R-CHOP), achieving complete remission, who presented nine years later with recurrent hyperlymphocytosis at 10.8 × 10^9^/L with villous appearance, massive splenomegaly, and systemic symptoms. Bone marrow evaluation demonstrated small-cell lymphoid infiltration, and flow cytometry showed CD19 positivity, CD5 negativity, strong FMC7 and CD11c expression, and a CD200/CD180 ratio of 0.3, consistent with SDRPL. Karyotype was normal. The patient was treated with rituximab-bendamustine and achieved complete remission. Case 2 was a 53-year-old man presenting for three months with splenic discomfort, systemic symptoms, and hyperlymphocytosis at 22.6 × 10^9^/L with villous morphology. SDRPL diagnosis was confirmed by combining bone marrow aspiration showing a small-cell lymphomatous process and flow cytometry showing CD19 positivity, CD5 negativity, strong FMC7 and CD11c expression, and a CD200/CD180 ratio of 0.5. Karyotype was normal. Rituximab monotherapy was followed by rapid improvement, with normalization of the lymphocyte count to 2.13 × 10^9^/L and regression of splenomegaly from the first cycle. These observations highlight the importance of combining lymphocyte immunophenotyping and bone marrow aspiration in isolated splenomegaly with villous lymphocytes on blood smear to avoid underdiagnosis of this new histopathological entity and therefore guide therapeutic management in the absence of guidelines.

## Introduction

Splenic diffuse red pulp small B-cell lymphoma (SDRPL) is a rare mature B-cell neoplasm that primarily involves the splenic red pulp and may extend to the bone marrow and peripheral blood [1,2]. Because only a limited number of cases and small series have been reported, its true incidence remains uncertain, although it appears to account for less than 1% of non-Hodgkin lymphomas [1]. The prognosis of this indolent B‑cell lymphoproliferative disorder is generally favorable when diagnosed early.

Clinically, SDRPL mainly manifests as massive splenomegaly, often causing abdominal discomfort. Systemic symptoms are described in one-third of patients, while lymph node involvement is rare and generally limited to the splenic hilum. Blood involvement is frequent [1-3].

Splenic histology remains the reference examination for diagnosis, revealing diffuse infiltration of the red pulp by monomorphic small B lymphocytes, effacing the white pulp; however, splenectomy is not always justified when less invasive investigations provide sufficiently concordant evidence [2,4,5].

In the absence of splenic histology, diagnosis can be suggested by bone marrow infiltration with a predominantly intrasinusoidal pattern, predominance of villous lymphocytes on peripheral blood smear with characteristic immunophenotype, and exclusion of other differential diagnoses, mainly splenic marginal zone lymphoma (SMZL) and hairy cell leukemia, which can also present with massive splenomegaly and villous lymphocytes [2,5].

​Lymphoma cells in SDRPL represent a clonal mature B-cell population that is generally CD5 negative, CD23 negative, and CD43 negative, as in SMZL, but CD23 aberrations are reported in some cases. They are distinguished by a more intense expression of CD11c and CD180 positivity, and the CD200/CD180 ratio has been reported as useful in the differential diagnosis with other splenic lymphomas. A low CD200/CD180 ratio (≤0.5) favors SDRPL, while a higher ratio supports SMZL or hairy cell leukemia [4,6,7]. The distinction is relevant because these entities may differ in their long‑term prognosis and, in some cases, in their management strategies, particularly compared with hairy cell leukemia, which requires specific regimens.

The underdiagnosis of SDRPL makes the development of therapeutic guidelines still poorly defined. This article aims, through the presentation of two observed cases, to illustrate how a multiparametric diagnostic approach can support the diagnosis of SDRPL in the absence of splenectomy and to report the therapeutic outcomes observed, thereby contributing to the literature on this rare entity.

## Case presentation

Case 1

A 65‑year‑old woman with no significant past medical history and good performance status, initially diagnosed in 2017 with SMZL and treated with R-CHOP with complete remission, presented eight years later (in September 2025) with recurrent systemic symptoms, including weight loss and night sweats, associated with massive splenomegaly. Blood count revealed hyperlymphocytosis at 10.8 × 10^9^/L (1.0-4.0 × 10^9^/L), associated with normocytic normochromic nonregenerative anemia at 9 g/dL (12-15 g/dL), normal platelet count at 185 × 10^9^/L (150-400 × 10^9^/L). The blood smear showed small monomorphic lymphocytes, round-to-oval nuclei with moderately condensed chromatin, and cytoplasm with cytoplasmic projections. Complementary lymphocyte immunophenotyping revealed a monotypic B-lymphoid population, CD19 positive, CD5 negative, CD23 negative, strong FMC7 positive, CD79b positive, weak CD200 positive, strong CD180 positive, strong CD11c positive, expressing kappa light chain of strong intensity estimated at 64% of lymphocytes. The CD200/CD180 ratio is 0.3, reflecting a low ratio (≤0.5) in favor of SDRPL, in contrast to SMZL.

**Figure 1:**
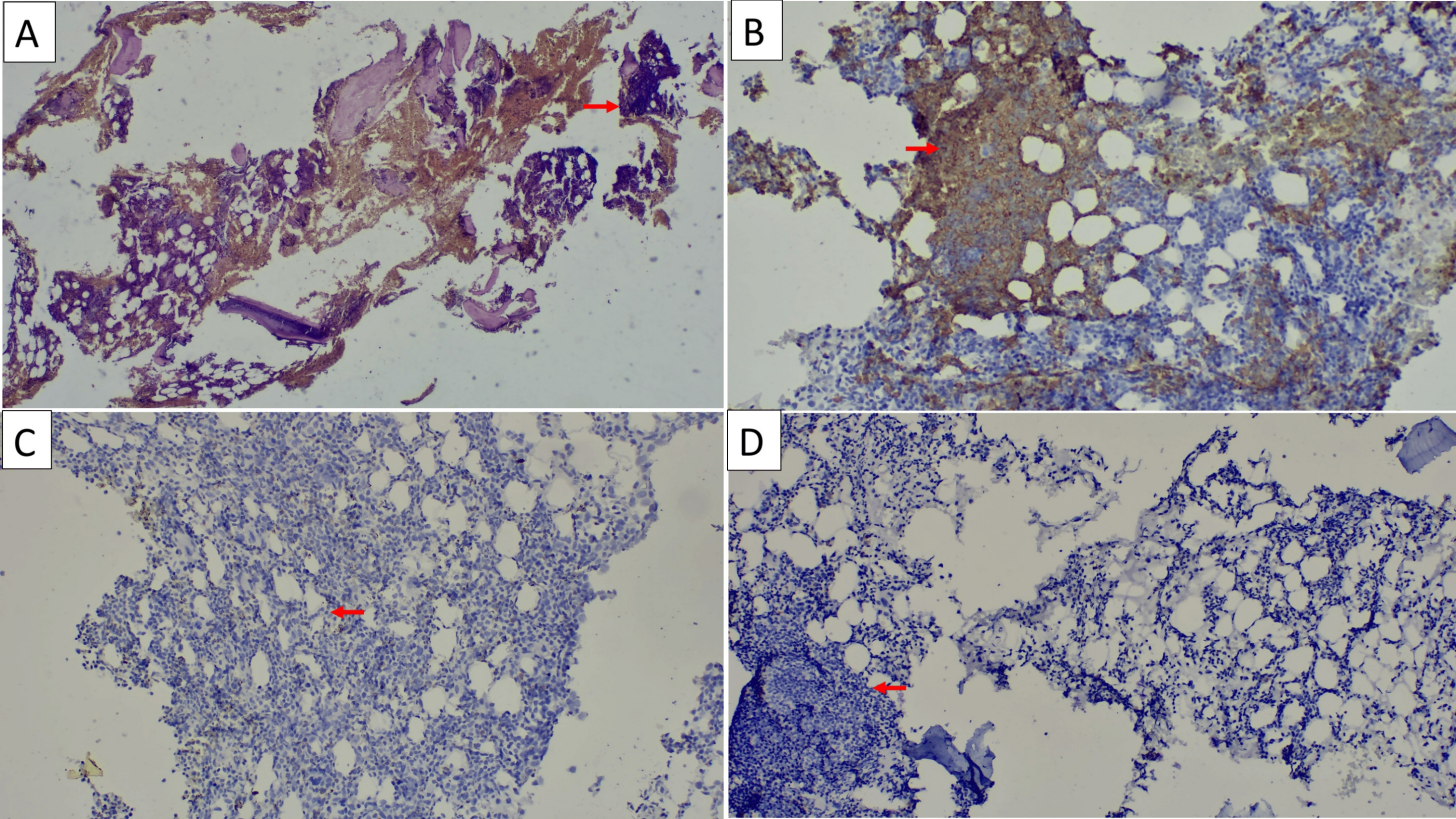
Interstitial bone marrow infiltration by SDRPL SDRPL: splenic diffuse red pulp small B-cell lymphoma; HES: hematoxylin and eosin stain (A) HES ×4: interstitial infiltration by small lymphoid cells (arrow shows representative example). (B) Anti-CD20 ×10: diffuse positivity (arrow shows representative example of CD20+ cells). (C) Anti-CD5 ×10: negative (arrow shows representative example of infiltrate lacking CD5). (D) Anti-CD23 ×10: negative (arrow shows representative example of infiltrate lacking CD23)

Bone marrow biopsy revealed interstitial infiltration by organized lymphocyte proliferation. Tumor cells are small-sized, with discrete cytonuclear atypia. The nucleus is enlarged, hyperchromatic, and surrounded by an irregular nuclear membrane. The cytoplasm is scant, with well-defined limits. Medullary fibrosis is minimal. Immunohistochemistry shows the following: CD20 positive, CD5 negative, p53 negative, Bcl2 negative, CD23 negative, and low Ki67, compatible with SDRPL (Figure 1).

**Figure 2:**
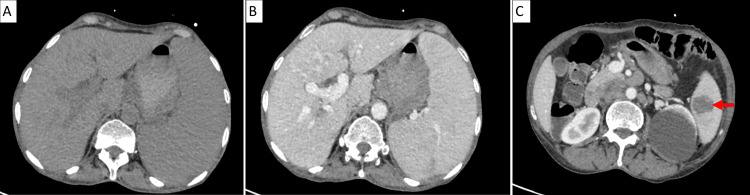
Abdominal sections from the CTCAP showing splenomegaly with a lower pole splenic tissue lesion CTCAP: chest-abdomen-pelvis computed tomographic scan (A) Transverse section without contrast. (B) Transverse section with contrast. (C) Transverse section with contrast centered on the lower pole tissue lesion (arrow)

The bone marrow karyotype revealed no chromosomal abnormalities. Extension workup by chest-abdomen-pelvis computed tomographic scan (CTCAP) revealed splenomegaly with a lower pole tissue lesion, associated with multiple para-aortic and celiac-mesenteric lymphadenopathies forming tissue sheaths (Figure 2).

The diagnosis was revisited, and the case was reclassified as SDRPL. The patient was put on second‑line rituximab 375 mg/m² on day 1 plus bendamustine 90 mg/m² on days 1 and 2, every 28 days, for a total of six cycles. Systemic symptoms and splenomegaly disappeared, and the lymphocyte count normalized to 1.15 × 10⁹/L.

Case 2

A 53‑year‑old man with no relevant medical history presented with three months of abdominal discomfort, associated with weight loss of 20 kg in one year, without night sweats or fever. Clinical examination found massive splenomegaly extending below the umbilicus, without palpable peripheral lymphadenopathy. Abdominal ultrasound showed massive splenomegaly with portal dilatation at 15 mm and homogeneous hepatomegaly. Blood count revealed hyperlymphocytosis at 22.6 × 10^9^/L (1.0-4.0 × 10⁹/L), associated with hypochromic microcytic anemia at 7.6 g/dL (13-17 g/dL) and thrombocytopenia at 97 × 10^9^/L (150-400 × 10^9^/L). Ferritin was slightly elevated at 197.02 ng/mL (13-150 ng/mL). The blood smear showed lymphocytosis consisting of small monomorphic lymphocytes with dense nuclei and villous cytoplasmic projections. Complementary lymphocyte immunophenotyping showed a monotypic B lymphoid population, CD19 positive, CD5 negative, CD23 positive, CD43 negative, strong FMC7 positive, weak CD79b positive, weak CD200 positive, strong CD180 positive, strong CD11c positive, expressing lambda light chain of strong intensity estimated at 80% of lymphocytes. The CD200/CD180 ratio is 0.5, which is low (≤0.5) and in favor of SDRPL, in contrast to SMZL or hairy cell leukemia.

**Figure 3:**
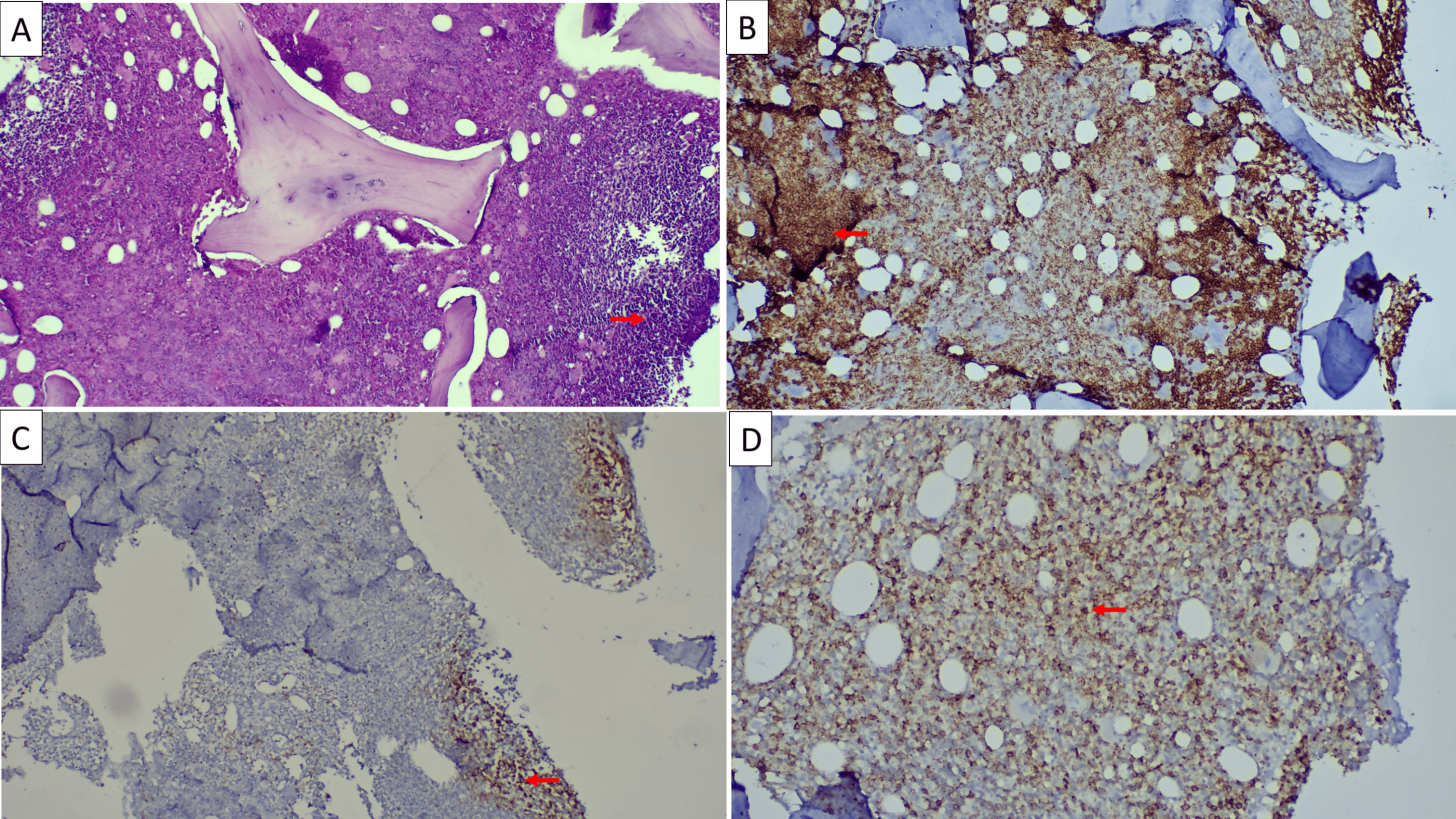
Diffuse bone marrow infiltration by SDRPL SDRPL: splenic diffuse red pulp small B-cell lymphoma; HES: hematoxylin and eosin stain (A) HES ×10: diffuse infiltration by sheets of small lymphoid cells (arrow shows representative example). (B) Anti‑CD20 ×20: diffuse positivity of tumor lymphoid cells (arrow highlights representative example of CD20+ cells). (C) Anti‑CD5 ×10: staining limited to reactive T lymphocytes (arrow indicates representative example of reactive T lymphocytes with CD5 positivity). (D) Anti‑CD23 ×40: diffuse positivity of the tumor B‑lymphoid population (arrow indicates representative example of CD23+ cells)

The bone marrow biopsy showed diffuse interstitial lymphoid infiltration, consisting of small monomorphic lymphocytes with round to oval nuclei and dense and compact chromatin. The cytoplasm was scant, with indistinct limits. Immunohistochemistry showed CD20 positive, CD10 negative, Bcl2 positive, CD5 negative, CD21 negative, and CD23 positive, compatible with medullary infiltration by SDRPL (Figure 3).

**Figure 4:**
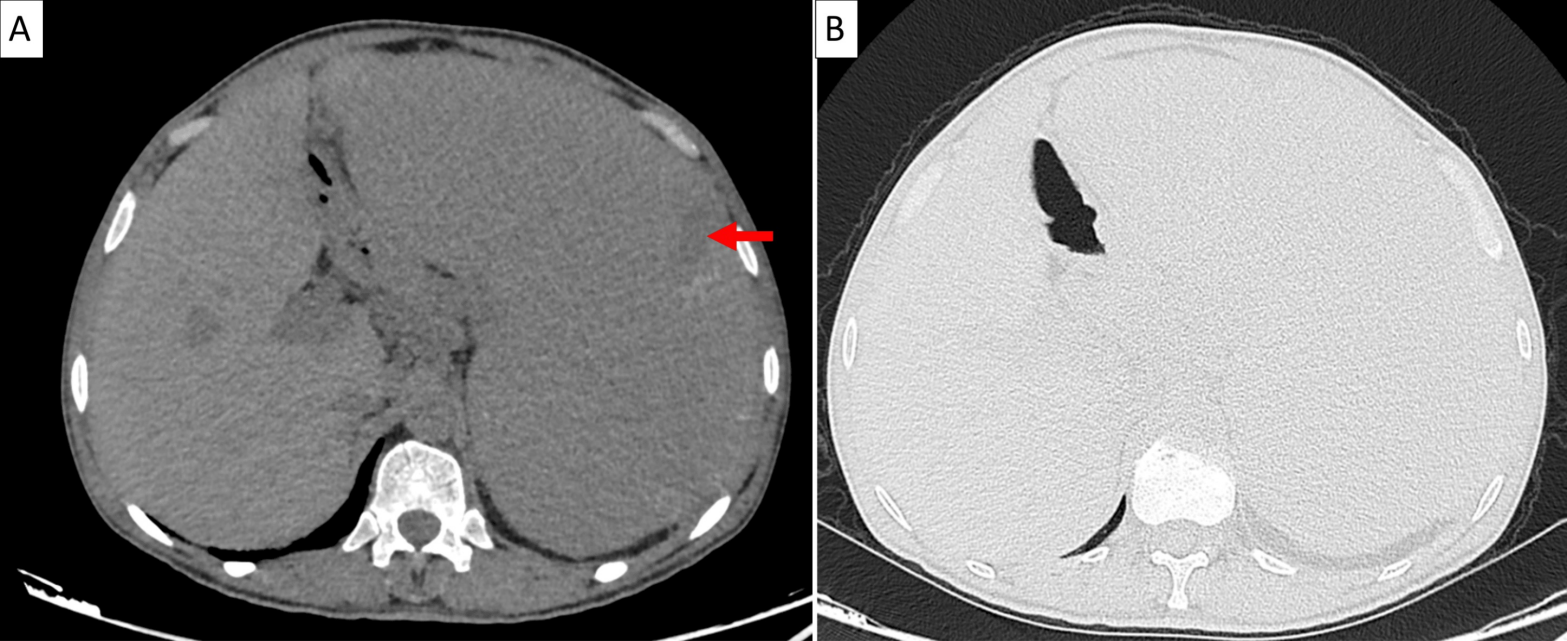
Abdominal sections from the CTCAP showing massive splenomegaly with a splenic infarction focus (arrow) CTCAP: chest-abdomen-pelvis computed tomographic scan (A) Transverse noncontrast section. (B) Transverse contrast-enhanced section

The bone marrow karyotype revealed no chromosomal abnormalities. Extension workup by CTCAP showed massive splenomegaly measuring 25 cm with hypodense areas related to infarction foci associated with subdiaphragmatic lymphadenopathies (Figure 4).

The patient was on first-line rituximab monotherapy at a dose of 375 mg/m² weekly, for a total of six cycles, with normalization of the lymphocyte count and regression of splenomegaly during follow‑up.

## Discussion

SDRPL was initially described among unclassifiable splenic B-cell lymphomas/leukemias and is now recognized as a distinct splenic small B-cell neoplasm in contemporary classifications [1,8]. Due to its rarity, only a few retrospective case series have detailed its clinicopathological characteristics [3,4]. The cases reported here seem to present similarities with cases reported in the literature: median age around 65 years (here 65 and 53 years), massive splenomegaly as the main symptom, and moderate lymphocytosis. Systemic symptoms are frequent and manifested in our two patients by weight loss, with associated night sweats in the first patient.

The splenectomy remains the gold standard for definitive diagnosis. The typical splenic histology of SDRPL is characterized by diffuse monomorphic lymphomatous infiltration in red pulp with white pulp atrophy [4,9]. In current practice, splenectomy should not be performed solely for diagnostic purposes when a set of less invasive findings is available [5]. Moreover, the majority of published SDRPL cases, including the seminal series [4,9], were diagnosed through retrospective reanalysis of archived splenectomy specimens, supporting the validity of our noninvasive multiparametric approach. Peripheral blood morphology, flow cytometry, and bone marrow histology can provide strong diagnostic support, particularly when the overall pattern is consistent with SDRPL [2,5,7].

In our observations, this multiparametric approach allowed reliable diagnosis without splenectomy. The first element supporting the diagnosis was immunophenotyping showing a monotypic B population, CD19 positive, CD5 negative, strong FMC7 positive, strong CD11c positive, and strong CD180 positive, with a CD200/CD180 ratio ≤0.5. The CD200/CD180 ratio is a highly discriminant marker of SDRPL. A low CD200/CD180 ratio (≤0.5) favors SDRPL, whereas a higher ratio (>0.5) is more consistent with SMZL or hairy cell leukemia variants [7]. The second element was medullary infiltration compatible with SDRPL: interstitial (case 1) or diffuse (a previously undescribed pattern suggestive of an advanced stage; case 2), without fibrosis, unlike SMZL, typically nodular [2,6].

These findings, taken together with the peripheral blood morphology and clinical presentation, favored SDRPL over SMZL and other splenic lymphoid neoplasms. The distinction between SDRPL and SMZL is complex due to an overlap of clinical and biological characteristics, as illustrated by the first patient initially diagnosed as SMZL in 2017. The main distinguishing points are summarized in Table 1 [6,7].

**Table 1:** Discriminant diagnostic criteria SMZL/SDRPL and comparison to our clinical cases SMZL: splenic marginal zone lymphoma; SDRPL: splenic diffuse red pulp small B-cell lymphoma

Criterion	SMZL	SDRPL	Case 1	Case 2
Peripheral villous lymphocytes	Heterogeneous distribution	Monomorphic distribution	Monomorphic distribution	Monomorphic distribution
Immunophenotyping	CD5-/+	CD5-	CD5-	CD5-
	CD23-	CD23-	CD23-	CD23+
	Low FMC7	Strong FMC7+	Strong FMC7+	Strong FMC7+
	Low CD11c	Strong CD11c+	Strong CD11c+	Strong CD11c+
	CD200/CD180 ratio >0.5	Low CD200+, strong CD180+, CD200/CD180 ratio ≤ 0.5	Low CD200+ , strong CD180+, CD200/CD180 ratio = 0.3	Low CD200+, strong CD180+, CD200/CD180 ratio = 0.5
Splenic histology	Marginal zone expansion (typical biphasic pattern)	Diffuse lymphomatous infiltration with red pulp congestion	Not performed (invasive)	Not performed (invasive)
Bone marrow biopsy	Intrasinusoidal and nodular infiltration, rarely interstitial	Interstitial or intrasinusoidal infiltration	Interstitial infiltration.	Diffuse infiltration
Cytogenetic abnormalities	80% (+3, +18, del7q +)	32% (+3, del7q)	None	None

Regarding CD23, typically negative in SDRPL, it was positive in case 2, an aberration reported in some cases, without questioning the diagnosis, given the CD200/CD180 ratio of ≤0.5, which strongly supports SDRPL. This highlights the utility of this score in helping distinguish between SMZL and SDRPL [4,7].

Both of our cases had a normal bone marrow karyotype, consistent with the majority of SDRPL series, where chromosomal abnormalities affect only one-third of cases (most often 7q deletion, trisomy 18, and partial 3q trisomy), unlike SMZL, where they exceed 80% [2].

Currently, no standard treatment recommendation exists for SDRPL. Active surveillance is preferred in asymptomatic patients. In symptomatic presentations, rituximab monotherapy and splenectomy constitute the most frequently reported first-line options, with chemotherapy reserved for refractory or progressive forms [2]. Our observations confirm this approach, with a good response to rituximab in the first line (case 2) and complete remission under rituximab-bendamustine in the second line in our relapsed patient. Although limited by a small sample size, further studies are needed to confirm the outcome of this new entity and its sensitivity to the proposed standard treatment.

## Conclusions

These two cases support the practical value of a multiparametric diagnostic approach to SDRPL combining peripheral blood morphology, CD200/CD180 flow cytometry ratio, and bone marrow examination, thereby avoiding diagnostic splenectomy in selected patients. They also illustrate that rituximab-based treatment may provide meaningful clinical benefit in symptomatic disease. Larger studies are needed to better define the diagnostic criteria and therapeutic strategy for this rare lymphoma.
